# Supplementary material for: Measuring the rate of NADPH consumption by glutathione reductase in the cytosol and mitochondria
Source: PLoS One. 2024 Dec 5;19(12):e0309886. doi: 10.1371/journal.pone.0309886 (PMC11620681; doi:10.1371/journal.pone.0309886)
Supplement: S1 File — (PDF) [file pone.0309886.s001.pdf]

Measuring the rate of NADPH consumption by glutathione reductase in the cytosol and mitochondria

Kenneth Ting[1], Eric Floro[2], Riley Dow[1], Jenny Jongstra-Bilen[1], Myron Cybulsky[3], Jonathan Rocheleau[3]

[1]Department of Immunology, University of Toronto, Toronto, ON M5S 1A8, Canada;
[2]Institute of Biomedical Engineering, University of Toronto, Toronto, ON M5S 1A8, Canada;
[3]Toronto General Hospital Research Institute, University Health Network, Toronto, ON M5G 1L7, Canada

Cristina Chua
Straive

Protocol Info: Kenneth Ting, Eric Floro, Riley Dow, Jenny Jongstra-Bilen, Myron Cybulsky, Jonathan Rocheleau . Measuring the rate of NADPH consumption by glutathione reductase in the cytosol and mitochondria. **protocols.io** **https://protocols.io/view/measuring-the-rate-of-nadph-consumption-by-glutath-dj2h4qb6**

Created: August 22, 2024

Last Modified: August 22, 2024

Protocol Integer ID: 106281

Keywords: Nicotinamide adenine dinucleotide phosphate, glutathione, glutathione disulfide, glutathione-disulfide reductase, macrophages, glucose-6-phosphate dehydrogenase, lipopolysaccharide, anisotropy, oxidized low-density lipoproteins, Apollo-NADP+ sensors.

Funders Acknowledgement:
Canadian Institutes of Health Research Grant
Grant ID: FDN154299
Canadian Institutes of Health Research Project Grant
Grant ID: DOL 409157
Natural Sciences and Engineering Research Council of Canada Research Tools and Instrumentation Grant
Grant ID: 2018-00846

Abstract

Background: NADPH is an essential co-factor supporting the function of enzymes that participate in both inflammatory and anti-inflammatory pathways in myeloid cells, particularly macrophages. Although individual NADPH dependent pathways are well characterized, how these opposing pathways are co-regulated to orchestrate an optimized inflammatory response is not well understood. To investigate this, techniques to track the consumption of NADPH need to be applied. Deuterium tracing of NADPH remains the gold standard in the field, yet this setup of mass-spectrometry is technically challenging and not readily available to most research groups. Furthermore, NADPH pools are compartmentalized in various organelles with no known membrane transporters, suggesting that NADPH-dependent pathways are regulated in an organelle specific manner. Conventional methods such as commercial kits are limited to quantifying NADPH in whole cells and not at the resolution of specific organelles. These limitations reflect the need for a novel assay that can readily measure the consumption rate of NADPH in different organelles.
Methods: We devised an assay that measures the consumption rate of NADPH by glutathione-disulfide reductase (GSR) in the mitochondria and the cytosol of RAW264.7 macrophage cell lines. RAW264.7 cells were transfected with Apollo-$NADP^+$ sensors targeted to the mitochondria or the cytosol, followed by the treatment of 2-deoxyglucose and diamide. Intravital imaging over time then determined GSR-dependent NADPH consumption in an organelle-specific manner.

Discussion: In lipopolysaccharide (LPS)-stimulated RAW264.7 cells, cytosolic and mitochondrial NADPH was consumed by GSR in a time-dependent manner. This finding was cross validated with a commercially
available NADPH kit that detects NADPH in whole cells. Loading of RAW264.7 cells with oxidized low-density lipoprotein followed by LPS stimulation elevated GSR expression, and this correlated with a more rapid drop in cytosolic and mitochondrial NADPH in our assay. The current limitation of our assay is applicability to transfectable cell lines, and higher expression of plasmid-encoded sensors relative to endogenous glucose-6-phosphate dehydrogenase.

Materials

Materials

Biological materials:

1. RAW264.7 Mφ cell line (ATCC, Catalog #:TIB-71)

Reagents:

1. 0.25% Trypsin/ 0.1% EDTA (Wisent, Catalog#:325-043 EL)
2. DMEM 1X with 4.5 g/L Glucose, L-Glutamine & Sodium Pyruvate (Wisent, Catalog#:319-005-CL)
3. Premium Fetal Bovie Serum (FBS) Endotoxin < 1 EU/ml (Wisent, Catalog#:091-150)
4. PBS 1X, w/o Calcium & Magnesium (Wisent, Catalog#:311-010 CL)
5. Geneticin™ Selective Antibiotic (G418 Sulfate) (50 mg/mL) (ThermoFisher, Catalog#: 10131035)
6. Amaxa® Cell Line Nucleofector® Kit V (LONZA, Catalog#: VCA-1003)
7. HBSS, no calcium, no magnesium, no phenol red (HBSS) 1X (ThermoFisher, Catalog#: 14175095)
8. Diamide (Sigma Aldrich, Catalog#: D3648)
9. 2-DG (Sigma Aldrich, Catalog#: D8375)
10. Mitochondrial mTurq2-Apollo-NADP+ (Addgene, Catalog#: 193303)
11. CytosolicmVenus-Apollo-NADP+ (Addgene, Catalog#: 71797)
12. Fluorescein(Sigma Aldrich, Catalog#: 46955)
13. BMHHbuffer (125 mm NaCl, 5.7 mm KCl, 2.5 mm CaCl2, 1.2 mm MgCl2, 10 mm HEPES, and 0.1% bovine serum albumin, pH 7.4)
14. Trypan Blue Solution (Sigma, Catalog#: T8154)

Solutions and Recipes:

1. BMHH imaging buffer (125 mM NaCl, 5.7 mM KCl, 2.5 mM CaCl2, 1.2 mM MgCl2, 0.1% bovine serum albumin (BSA), and 10 mM 4-(2-hydroxyethyl)-1-piperazineethanesulfonic acid (HEPES) at pH 7.4).

Laboratory supplies and equipment:

1. Hemocytometer cell counting chamber (Sigma, Catalog#: BR719505-1EA)
2. Lonza™ Nucleofector™ Transfection 2b Device (Fisher Scientific, Catalog#: 13458999)
3. T75 tissue culture treated flask (StemCell Technologies, Catalog#: 200-0501)
4. T25 tissue culture treated flask (Sigma, Catalog#: C6356-200EA)
5. 35 mm Dish (No. 1.5 Coverslip, 14 mm Glass Diameter, Uncoated) (Mattek, Catalog#: P35G-1.5-14-C)
6. Custom-built wide-field RAMM microscope (ASI) equipped with excitation light-emitting diodes (LEDs) (405, 505, and 590 nm) and an excitation polarizing filter (Edmund Optics, Barrington, NJ), and built to perform simultaneous two-color fluorescence anisotropy imaging.
7. Images were collected using 40×/0.75 NA air objective lens (Olympus, Richmond Hill, Canada).
8. Fluorescence was passed through a Cerulean (ET470/24M) or Venus (ET535/30M) emission filter on a filter wheel, and subsequently split using an Optosplit II (Cairn, Faversham, U.K.) to simultaneously collect parallel and perpendicular emission light on separate regions of an IRIS 15 CMOS camera (Teledyne Photometrics, Tucson, AZ). Excitation was also passed through a triple band filter containing ET-350/55-470/30-557/35 band-pass filters.

Part I: Transfection of Apollo-NADP+ sensors in RAW264.7 Mφ cell lines

1 One week prior to nucleofection, passage cells in 15mL of DMEM media supplemented with 10% FBS for every 2-3 days in a T75 tissue culture treated flask.

2 To passage cells, remove cultured media and treat cells with 3mL of trypsin for 5 minutes at 37°C/5% $CO2$ for detachment. Next, add 3mL of fresh DMEM media to neutralize trypsin and centrifuge the cells at 90xg for 5 minutes.

3 Remove the supernatant, resuspend the cells with 10mL of fresh DMEM media, quantify the number of cells using a hemocytometer cell counting chamber and passage them at a ratio of 1:5 to 1:8.

4 One day prior to nucleofection, passage the cells at a ratio of 1:3.

5 On the day of nucleofection, retrieve the nucleofector solution and supplement from Amaxa® Cell Line Nucleofector® Kit V. For the first time usage, ensure to add all the supplement to the solution

6 Fill one well of a 6-well plate with 1 mL of DMEM that is supplemented with 20% FBS. Pre-warm the plate at 37°C/5% $CO2$.

7 Centrifuge and isolate 2 x 106 cells. Remove supernatant and rinse cells with 1mL of pre-warmed 1X PBS. Repeat centrifugation, remove PBS and resuspend pellet with 100 µl of supplemented nucleofector solution. Add 5 µg of mitochondrial mTurq2-Apollo-NADP+ sensor or cytosolic mVenus-Apollo-NADP+ sensor to the mixture.

8 Transfer the complete mixture to kit-supplied cuvette without the injection of bubbles. Insert the cuvette into a Nucleofector® II Device and select program D-032 by pressing the X-button. Upon completion of nucleofection, retrieve the cuvette and add 500 µl of pre-warmed DMEM supplemented with 20% FBS into the suspension.

9 Transfer the entire 600ul of suspension using kit-supplied pipettes into the pre-warmed 6 well-plate in a drop-wise manner (total volume is 6 mL in the well).

10 3 hours after the cells have adhered, replace the cultured media with 2mL of fresh DMEM media supplemented with 20% FBS for recovery.

11 When cell confluency has reached a minimum of 80% (usually 48 hours post transfection), the cells can be replated for experiments where transient nucleofection of the sensor is desired.

12 For stable selection, re-seed all the transfected cells from step 11 to a T25 culture flask with 5mL of DMEM media supplemented with 10% FBS. 3 hours after the cells have adhered,

replace the cultured media with fresh DMEM media supplemented with 10% FBS and G418 Sulfate at final concentration of 400 µg/ml.

13 Replace the media with fresh 5mL of DMEM media supplemented with 10% FBS and G418 for every 2-3 days until one week. If cell confluency has reached a minimum of 80% in the T25 flask, re-seed the cells to a T75 flask or 10 cm tissue culture treated dish with 10mL of DMEM media supplemented with 10% FBS and G418.

14 To set up imaging of the sensors, isolate and seed 1x106 cells in a series of 35-mm petri dish (one dish per time point of the kinetic) with 2mL of DMEM media supplemented with 10% FBS and G418 one day prior to imaging.

Part II: Imaging of the Apollo-NADP+ sensors in RAW264.7 Mφ cell lines

15 On the day of imaging, prepare a 1:1 mixture of diamide (250 mM) and 2-DG (250 mM) in HBSS buffer. Specifically, add 1mL of 500 mM of diamide with 1mL of 500 mM of 2-DG.

16 On the day of imaging, prepare and calibrate microscope for imaging of Apollo-NADP+ sensor.

17 Turn on microscope at least 30min prior to any imaging to ensure CMOS camera temperature reaches equilibrium (Iris 15 camera was used in this case).

18 Launch microscope management software, in this case open source software Micro-Manage was used (available for download at https://micro-manager.org/Download_Micro-Manager_Latest_Release)

19 Set binning. In this case binning was set to 4x4 to collect the maximum image intensity.

20 Live imaging parameters should be set low to limit photobleaching and phototoxicity. In this case LED and exposure time were set to 20% and ms, respectively.

21 Ensure LED is properly aligned (i.e., evenly illuminates the back aperture of the lens) by first turning on the YFP LED, then holding a piece of paper against the microscope stage adjust the LED position until the emitted light appearing on the paper becomes sharp.

22 Calibrate polarizer by first ensuring the polarizer is placed in the excitation path.

23 Next, place a YFP-autofluorescent-plastic-slide on the microscope stage.

24 Begin live imaging at low intensity (20%) and low exposure time (10-50 ms), then using the draw tool draw a box around the live image of both the parallel and perpendicular channels.

25 Then select 'Analyze' > 'Plot Profile'. This will display a live profile of the intensity of both the parallel and perpendicular channels.

26 Finally, adjust the polarizer until the difference in intensity values between the parallel and perpendiculr channels is maximized. When done correctly the parallel channel will have an overall higher intensity than perpendicular.

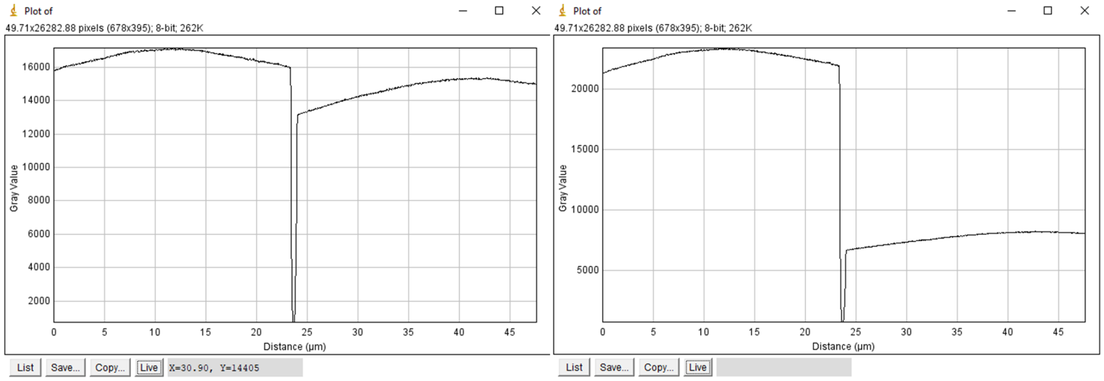

Figure 1: For step d,iv. Left: incorrectly adjusted polarizer.
Right: Correctly adjusted polarizer

27 Remove the cultured media and replace it with 900µl of pre-warmed HBSS.

28 Determine image acquisition parameters, LED intensity (%) and exposure time (ms).

29 Place cells in pre-warmed HBSS onto stage top and use brightfield settings locate and focus on the cells.

30 Switch to fluorescence imagine (e.g., YFP imaging), and use live imaging settings to fine focus on the cells/sensor. Set LED intensity to 80% and experiment with different image acquisition exposure times to find parameters that produce final sensor intensities within your camera's known saturation levels. In this case the total intensity in the parallel channel was roughly 40,000 AU, which was commonly achieved with an LED intensity of 80% and exposure time of 800 ms.

31 For the first time point, do not add any diamide and 2-DG. Image the sensor to acquire the baseline value of the control and experimental condition. TROUBLESHOOTING: Please refer to General Notes and Troubleshooting if the sensors were not expressed or weakly expressed.

32 For the second time point, add 100µl of the 1:1 mixture of diamide and 2-DG into the first petri dish, such that the final concentration of diamide and 2-DG is 25mM respectively. Incubate for 5 minutes, followed by imaging of the sensors.

33 For the third and subsequent time points, repeat step 6 for an additional 5 minute-interval until the completion of the kinetics.

34 At the end of imaging acquire images of standard anisotropy fluorescein solution for instrument diagnostics and G-factor correction.

35 Start by preparing a solution of 5 mM fluorescein dissolved in BMHH.

36 Set LED intensity and exposure time such that the acquired image has roughly the same total intensity of the sensor in cells, in this case 40,000 AU at 20% LED and 20 ms exposure time.

37 Fluorescein solution can be left in the fridge at 4°C for 1 month for repeated uses.

Part IV: Data analysis

38 Parallel (I∥) and perpendicular (I⊥) fluorescence intensity images were analyzed with a custom ImageJ plugin. These are available at https://github.com/RocheleauLab/Optosplit-Anisotropy-Analysis-scripts. The following three imageJ macro scripts are required: 00-Preporcessing, 01-Anisotropy Macro, 03-ROI Selection

39 The images were background-corrected using a rolling ball filter where the radius is larger than the largest cell in your images but small enough that distortions in the background are removed, in this case a radius of 100 was used. Pixel-by-pixel anisotropy (r) was calculated using the background-corrected intensities: $r = (I_{\parallel} - GI_{\perp})/(I_{\parallel} + 2G_{\perp})$.

40 Start by navigating to the folder containing all images and create the following two folders: GfactorImages, AlignmentImages

41 Drag and drop your fluorescein image into the GfactorImages folder.

42 Select an image of your cells and drag and drop into the imageJ application.

43 On the imageJ application select Plugins > Macros > Record...

44 Using the draw Rectangle tool, draw two boxes of the same size and position on the parallel and perpendicular channels of the image

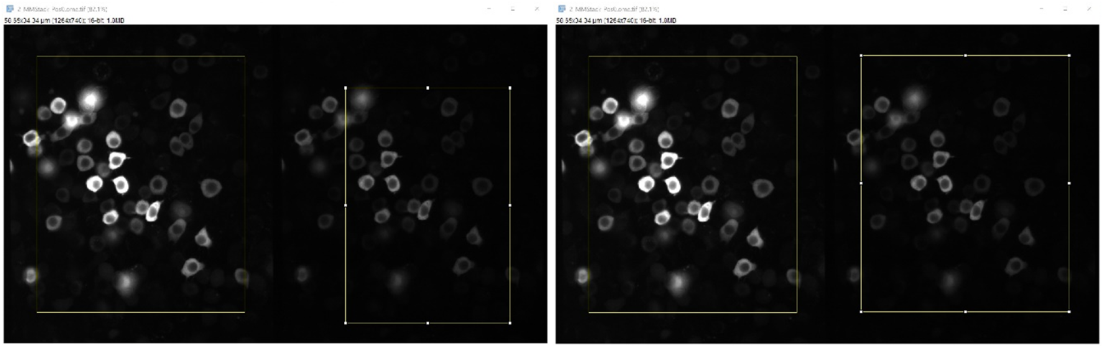

Figure 2: For step c,ii. Left: incorrectly drawn boxes, right: correctly drawn boxes

45 Dimensions for the boxes drawn will appear in the Record dialogue box. Copy and paste these dimensions into 00-Preprocessing script under rect_para and rect_perp

46 Run 00-Preprocessing script

47 A file explore dialogue box will open. Navigate to Alignment Folder > Transform "select"

48 Another file explore dialogue box will open. This time navigate to Alignment Folder > Original > and "select" the image titled "Para"

49 The script will continue running through all the images

50 Processed images will be placed in a new folder titled "T2_Processed"

51 The script will continue running through all images in the experiment folder. This may take several minutes and depends on the number of images acquired

52 Gfactor for the wide-field microscope was calculated using fluorescein solutions, which have an anisotropy near zero, thus simplifying the standard anisotropy equation to $G = I\parallel/I\perp$.

53 After 00-Preprocessing script has finished running, navigate to 01-Anisotropy Macro script and press Run

54 A file explorer dialogue box will open, select the experiment folder you are working with

55 Allow the script to run. This may take several minutes depending on the number of images acquired

56 Regions of interest (ROI) were selected using the parallel intensity images to avoid selection bias.

57 After 01-Anisotropy Macro script has finished, navigate to 03-ROI Selection and press Run

58 Using either the oval tool or the 'any shape tool' draw regions of interest over the largest portion of where the sensor is appearing in the Para channel

59 Each independent trial consists of at least five to more than 30 cells (5–30 technical replicates).
