# Supplementary figures and images for: Measuring the rate of NADPH consumption by glutathione reductase in the cytosol and mitochondria
Source: PLoS One. 2024 Dec 5;19(12):e0309886. doi: 10.1371/journal.pone.0309886 (PMC11620681; doi:10.1371/journal.pone.0309886)

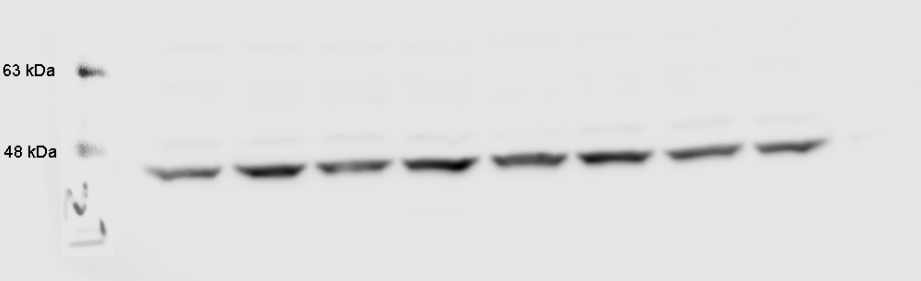

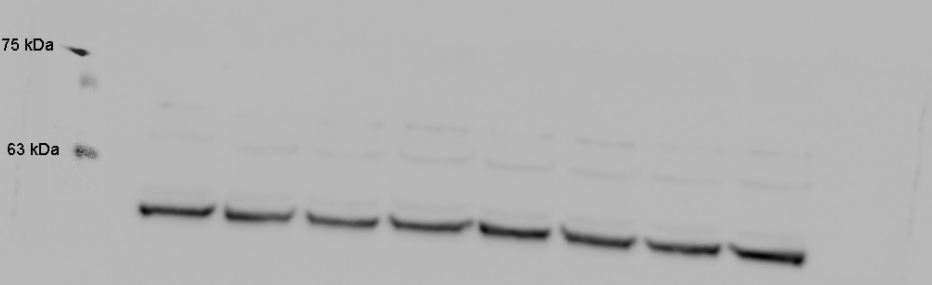

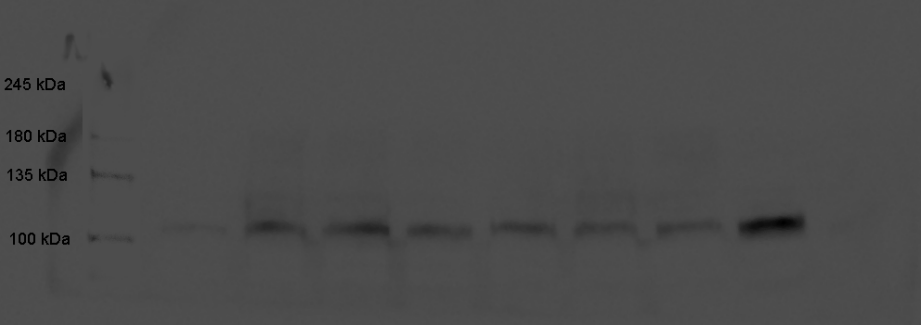

Supplement: S2 File — (PDF) [file pone.0309886.s002.pdf]
