# Supplementary material for: Measuring the rate of NADPH consumption by glutathione reductase in the cytosol and mitochondria
Source: PLoS One. 2024 Dec 5;19(12):e0309886. doi: 10.1371/journal.pone.0309886 (PMC11620681; doi:10.1371/journal.pone.0309886)
Supplement: Attachment — Submitted filename: PONE-D-24-18553 reviewer.pdf [file pone.0309886.s003.pdf]

PLOS ONE

Measuring the rate of NADPH consumption by glutathione reductase in the cytosol and mitochondria

--Manuscript Draft--

**Manuscript Number:**	PONE-D-24-18553
**Article Type:**	Lab Protocol
**Full Title:**	Measuring the rate of NADPH consumption by glutathione reductase in the cytosol and mitochondria
**Short Title:**	Measuring cytosolic and mitochondrial NADPH consumption rate by glutathione reductase
**Corresponding Author:**	Myron Cybulsky<br>TGH: Toronto General Hospital<br>Toronto, ON CANADA
**Keywords:**	Nicotinamide adenine dinucleotide phosphate, glutathione, glutathione disulfide, glutathione-disulfide reductase, macrophages, glucose-6-phosphate dehydrogenase, lipopolysaccharide, anisotropy, oxidized low-density lipoproteins, Apollo-NADP+ sensors.
**Abstract:**	Background: NADPH is an essential co-factor supporting the function of enzymes that participate in both inflammatory and anti-inflammatory pathways in myeloid cells, particularly macrophages. Although individual NADPH-dependent pathways are well characterized, how these opposing pathways are co-regulated to orchestrate an optimized inflammatory response is not well understood. To investigate this, techniques to track the consumption of NADPH need to be applied. Deuterium tracing of NADPH remains the gold standard in the field, yet this setup of mass-spectrometry is technically challenging and not readily available to most research groups. Furthermore, NADPH pools are compartmentalized in various organelles with no known membrane transporters, suggesting that NADPH-dependent pathways are regulated in an organelle-specific manner. Conventional methods such as commercial kits are limited to quantifying NADPH in whole cells and not at the resolution of specific organelles. These limitations reflect the need for a novel assay that can readily measure the consumption rate of NADPH in different organelles.<br>Methods: We devised an assay that measures the consumption rate of NADPH by glutathione-disulfide reductase (GSR) in the mitochondria and the cytosol of RAW264.7 macrophage cell lines. RAW264.7 cells were transfected with Apollo-NADP+ sensors targeted to the mitochondria or the cytosol, followed by the treatment of 2-deoxyglucose and diamide. Intravital imaging over time then determined GSR-dependent NADPH consumption in an organelle-specific manner.<br>Discussion: In lipopolysaccharide (LPS)-stimulated RAW264.7 cells, cytosolic and mitochondrial NADPH was consumed by GSR in a time-dependent manner. This finding was cross validated with a commercially available NADPH kit that detects NADPH in whole cells. Loading of RAW264.7 cells with oxidized low-density lipoprotein followed by LPS stimulation elevated GSR expression, and this correlated with a more rapid drop in cytosolic and mitochondrial NADPH in our assay. The current limitation of our assay is applicability to transfectable cell lines, and higher expression of plasmid-encoded sensors relative to endogenous glucose-6-phosphate dehydrogenase.
**Order of Authors:**	Kenneth Kin Yan Ting
	Eric Floro
	Riley Dow
	Jenny Jongstra-Bilen
	Myron Cybulsky
	Jonathan Rocheleau
**Additional Information:**	
**Question**	**Response**

Powered by Editorial Manager® and ProduXion Manager® from Aries Systems Corporation

**Financial Disclosure**<br><br>Enter a financial disclosure statement that describes the sources of funding for the work included in this submission. Review the submission guidelines for detailed requirements. View published research articles from *PLOS ONE* for specific examples.<br><br>This statement is required for submission and **will appear in the published article** if the submission is accepted. Please make sure it is accurate.<br><br>**Funded studies**<br>Enter a statement with the following details:<br>• Initials of the authors who received each award<br>• Grant numbers awarded to each author<br>• The full name of each funder<br>• URL of each funder website<br>• Did the sponsors or funders play any role in the study design, data collection and analysis, decision to publish, or preparation of the manuscript?<br><br>Did you receive funding for this work?	Yes
Please add funding details.<br>as follow-up to "**Financial Disclosure**<br><br>Enter a financial disclosure statement that describes the sources of funding for the work included in this submission. Review the submission guidelines for detailed requirements. View published research articles from *PLOS ONE* for specific examples.<br><br>This statement is required for submission and **will appear in the published article** if the submission is accepted. Please make sure it is accurate.	This work was supported by the Canadian Institutes of Health Research Grant FDN154299 (to M.I.C.). Additional support of this work was from Canadian Institutes of Health Research Project Grant DOL 409157 (to J.R.), and by Natural Sciences and Engineering Research Council of Canada Research Tools and Instrumentation Grant 2018-00846 (to J.R.). M.I.C. holds a Tier 1 Canada Research Chair. K.K.Y.T. was supported by fellowships from the Canadian Institutes of Health Research, Ontario Graduate Scholarship, fellowships from University of Toronto and The Peterborough K. M. Hunter Charitable Foundation. All sponsors did not play any role in the study design, data collection and analysis, decision to publish, or preparation of the manuscript.

Powered by Editorial Manager® and ProduXion Manager® from Aries Systems Corporation

**Funded studies**<br>Enter a statement with the following details:<br>• Initials of the authors who received each award<br>• Grant numbers awarded to each author<br>• The full name of each funder<br>• URL of each funder website<br>• Did the sponsors or funders play any role in the study design, data collection and analysis, decision to publish, or preparation of the manuscript?<br><br>Did you receive funding for this work?"	
Please select the country of your main research funder (please select carefully as in some cases this is used in fee calculation).<br>as follow-up to "**Financial Disclosure**<br><br>Enter a financial disclosure statement that describes the sources of funding for the work included in this submission. Review the submission guidelines for detailed requirements. View published research articles from *PLOS ONE* for specific examples.<br><br>This statement is required for submission and **will appear in the published article** if the submission is accepted. Please make sure it is accurate.<br><br>**Funded studies**<br>Enter a statement with the following details:<br>• Initials of the authors who received each award<br>• Grant numbers awarded to each author<br>• The full name of each funder<br>• URL of each funder website<br>• Did the sponsors or funders play any role in the study design, data collection and analysis, decision to publish, or preparation of the manuscript?<br><br>Did you receive funding for this work?"	CANADA - CA
**Competing Interests**	The authors have declared that no competing interests exist.

Use the instructions below to enter a competing interest statement for this submission. On behalf of all authors, disclose any competing interests that could be perceived to bias this work—acknowledging all financial support and any other relevant financial or non-financial competing interests.<br><br>This statement is required for submission and **will appear in the published article** if the submission is accepted. Please make sure it is accurate and that any funding sources listed in your Funding Information later in the submission form are also declared in your Financial Disclosure statement.<br><br>View published research articles from *PLOS ONE* for specific examples.<br><br>**NO authors have competing interests**<br><br>Enter: *The authors have declared that no competing interests exist.*<br><br>**Authors with competing interests**<br><br>Enter competing interest details beginning with this statement:<br><br>*I have read the journal's policy and the authors of this manuscript have the following competing interests: [insert competing interests here]*<br><br>* typeset	
**Ethics Statement**<br><br>Enter an ethics statement for this submission. This statement is required if the study involved:<br><br>• Human participants<br>• Human specimens or tissue<br>• Vertebrate animals or cephalopods<br>• Vertebrate embryos or tissues	N/A

Powered by Editorial Manager® and ProduXion Manager® from Aries Systems Corporation

• Field research<br><br>Write "N/A" if the submission does not require an ethics statement.<br><br>General guidance is provided below. Consult the submission guidelines for detailed instructions. **Make sure that all information entered here is included in the Methods section of the manuscript.**	

**Format for specific study types**<br><br>**Human Subject Research (involving human participants and/or tissue)**<br>• Give the name of the institutional review board or ethics committee that approved the study<br>• Include the approval number and/or a statement indicating approval of this research<br>• Indicate the form of consent obtained (written/oral) or the reason that consent was not obtained (e.g. the data were analyzed anonymously)<br><br>**Animal Research (involving vertebrate animals, embryos or tissues)**<br>• Provide the name of the Institutional Animal Care and Use Committee (IACUC) or other relevant ethics board that reviewed the study protocol, and indicate whether they approved this research or granted a formal waiver of ethical approval<br>• Include an approval number if one was obtained<br>• If the study involved *non-human primates*, add *additional details* about animal welfare and steps taken to ameliorate suffering<br>• If anesthesia, euthanasia, or any kind of animal sacrifice is part of the study, include briefly which substances and/or methods were applied<br><br>**Field Research**<br><br>Include the following details if this study involves the collection of plant, animal, or other materials from a natural setting:<br>• Field permit number<br>• Name of the institution or relevant body that granted permission	
**Data Availability**<br><br>Authors are required to make the data underlying their research findings fully available, without restriction.<br><br>If the manuscript reports sample data generated using the protocol, the data underlying those results must be made available at the time of publication. A Data	Yes - sample data are fully available without restriction

Availability Statement describing where the data can be found is required at submission. When legal or ethical requirements prohibit public data sharing, authors must indicate how researchers may obtain access to the data. See the PLOS Data Policy and FAQ for detailed information.<br><br>A Data Availability Statement describing where the data can be found is required at submission. Your answers to this question constitute the Data Availability Statement and **will be published in the article**, if accepted.<br><br>**Important:** Stating 'data available on request from the author' is not sufficient. If your data are only available upon request, select 'No' for the first question and provide justification in the text box.<br><br>For protocols without sample data or results, you must state this in your Data Availability Statement.<br><br>Do the authors confirm that EITHER the protocol does not report results OR that sample data will be made available?	
**Describe where the data may be found in full sentences. If you are copying our sample text, replace any instances of XXX with the appropriate details.**<br><br>• If the data are **held or will be held in a public repository**, include URLs, accession numbers or DOIs. If this information will only be available after acceptance, indicate this by ticking the box below. For example: *All XXX files are available from the XXX database (accession number(s) XXX, XXX.*).<br>• If the data are all contained **within the manuscript and/or Supporting Information files**, enter the following: *All relevant data are within the*	All relevant data are within the manuscript and its Supporting Information files.

Powered by Editorial Manager® and ProduXion Manager® from Aries Systems Corporation

manuscript and its Supporting Information files.<br>• If neither of these applies but you are able to provide **details of access elsewhere**, with or without limitations, please do so. For example:<br><br>*Data cannot be shared publicly because of [XXX]. Data are available from the XXX Institutional Data Access / Ethics Committee (contact via XXX) for researchers who meet the criteria for access to confidential data.*<br><br>*The data underlying the results presented in the study are available from (include the name of the third party and contact information or URL).*<br>• This text is appropriate if the data are owned by a third party and authors do not have permission to share the data.<br><br>* typeset	
Additional data availability information:	

Powered by Editorial Manager® and ProduXion Manager® from Aries Systems Corporation

Manuscript

Click here to access/download;Manuscript;PLOS ONE Lab protocol manuscript.docx

Title: Measuring the rate of NADPH consumption by glutathione reductase in the cytosol and mitochondria

Authors: Kenneth K.Y. Ting[1,3], Eric Floro[2,3], Riley Dow[1,3], Jenny Jongstra-Bilen[1,3,5], Myron I. Cybulsky[1,3,5,6*], Jonathan V. Rocheleau[2,3,4,7*]

Affiliations:

[1]Department of Immunology, University of Toronto, Toronto, ON M5S 1A8, Canada

[2]Institute of Biomedical Engineering, University of Toronto, Toronto, ON M5S 1A8, Canada

[3]Toronto General Hospital Research Institute, University Health Network, Toronto, ON M5G 1L7, Canada

[4]Department of Physiology, University of Toronto, Toronto, ON M5S 1A8, Canada

[5]Department of Laboratory Medicine and Pathobiology, University of Toronto, Toronto, ON M5S 1A8, Canada

[6]Peter Munk Cardiac Centre, University Health Network, Toronto, ON M5G 2N2, Canada

[7]Banting and Best Diabetes Centre, University of Toronto, Toronto, ON M5G 2C4, Canada

*Correspondence: myron.cybulsky@utoronto.ca; jon.rocheleau@utoronto.ca

Abstract

Background: NADPH is an essential co-factor supporting the function of enzymes that participate in both inflammatory and anti-inflammatory pathways in myeloid cells, particularly macrophages. Although individual NADPH-dependent pathways are well characterized, how these opposing pathways are co-regulated to orchestrate an optimized inflammatory response is not well understood. To investigate this, techniques to track the consumption of NADPH need to be applied. Deuterium tracing of NADPH remains the gold standard in the field, yet this setup of mass-spectrometry is technically challenging and not readily available to most research groups. Furthermore, NADPH pools are compartmentalized in various organelles with no known membrane transporters, suggesting that NADPH-dependent pathways are regulated in an organelle-specific manner. Conventional methods such as commercial kits are limited to quantifying NADPH in whole cells and not at the resolution of specific organelles. These limitations reflect the need for a novel assay that can readily measure the consumption rate of NADPH in different organelles.

Methods: We devised an assay that measures the consumption rate of NADPH by glutathione-disulfide reductase (GSR) in the mitochondria and the cytosol of RAW264.7 macrophage cell lines. RAW264.7 cells were transfected with Apollo-$NADP^+$ sensors targeted to the mitochondria or the cytosol, followed by the treatment of 2-deoxyglucose and diamide. Intravital imaging over time then determined GSR-dependent NADPH consumption in an organelle-specific manner.

Discussion: In lipopolysaccharide (LPS)-stimulated RAW264.7 cells, cytosolic and mitochondrial NADPH was consumed by GSR in a time-dependent manner. This finding was cross validated with a commercially available NADPH kit that detects NADPH in whole cells. Loading of RAW264.7 cells with oxidized low-density lipoprotein followed by LPS stimulation elevated GSR expression, and this correlated with a more rapid drop in cytosolic and mitochondrial NADPH in

our assay. The current limitation of our assay is applicability to transfectable cell lines, and higher expression of plasmid-encoded sensors relative to endogenous glucose-6-phosphate dehydrogenase.

Keywords

Nicotinamide adenine dinucleotide phosphate, glutathione, glutathione disulfide, glutathione-disulfide reductase, macrophages, glucose-6-phosphate dehydrogenase, lipopolysaccharide, anisotropy, oxidized low-density lipoproteins, Apollo-NADP$^+$ sensors.

Background

NADPH, the reduced form of nicotinamide adenine dinucleotide phosphate (NADP$^+$), is an essential co-factor for enzymes that catalyze a wide variety of oxidative and antioxidative processes (1). Notably in myeloid cells, the importance of NADPH is indisputable for driving their effector functions. For instance, upon stimulation of myeloid cells, NADPH is significantly depleted due to consumption by inflammatory and anti-inflammatory pathways (2-5). Although these individual pathways have been traditionally well-defined, how NADPH-dependent inflammatory and anti-inflammatory processes co-regulate each other to fine-tune an optimized inflammatory response is not well understood. Recently, we proposed that the competition for NADPH consumption between inflammatory and anti-inflammatory pathways is a naturally evolved mechanism that regulates the magnitude of an inflammatory response in immune cells (6). To test this hypothesis, the consumption of NADPH needs to be accurately tracked and determined. Although the use of targeted metabolite tracing, such as deuterium tracing of NADPH (7), is successful and remains the gold standard in the field, this approach requires mass-spectrometry, which may not be accessible to many laboratories. More importantly, conventional commercial kits that quantify NADPH and NADP$^+$ only measure the steady-state total abundance and do not provide information on the direction of the metabolic flux (i.e., the rates of consumption vs. synthesis). Therefore, taken together, these limitations reflect the need for developing a more accessible and simpler assay that can measure the rate of NADPH consumption by a specific pathway, such as the one mediated by glutathione-disulfide reductase (GSR).

GSR is an anti-oxidative/anti-inflammatory enzyme that reduces glutathione disulfide (GS-SG) to glutathione (GSH) in a NADPH-dependent manner. GSR is conserved across eukaryotes and prokaryotes, with the exception of Gram-positive bacteria, some Gram-negative bacteria, certain insects, and parasites (8-11). This conservation is not surprising as oxidative stress is an inevitable byproduct of aerobic respiration, and GSH is an important antioxidant that mitigates the effects of reactive oxygen species (ROS), such as hydrogen peroxide. As expected, GSR tends to be localized in regions of the cell where a high electron flux is located (12). For instance, in prokaryotes, GSR is found in the periplasm (13, 14), while in eukaryotes, it is found in the mitochondria, cytosol, endoplasmic reticulum and nucleus (15-17). Thus, GSR is critical for regenerating GSH and maintaining a favorable redox environment. GSR enzymatic activity requires NADPH as a co-factor, which consumes NADPH by converting it to NADP$^+$. Since the consumption of NADPH by GSR may compete with NADPH-dependent inflammatory processes, we endeavored to determine NADPH consumption by GSR in macrophages (Mφs) stimulated by lipopolysaccharide (LPS), a classical Toll-like receptor 4 ligand.

Here, we describe a novel kinetic assay to measure NADPH consumption rate by GSR in the cytosol and the mitochondria (Fig 1A). This technique has been validated and applied in our recently published study (6). Because accurate determination of the NADPH consumption rate can be confounded by the reduction rate of $NADP^+$, we inhibited key biochemical pathways that reduce $NADP^+$ back to NADPH in LPS-stimulated Mφs, including glucose-6-phosphate dehydrogenase (G6PD), isocitrate dehydrogenases and folate-mediated one carbon metabolism can contribute to the reduction of $NADP^+$ (18-20). We added 2-deoxyglucose (2-DG) in our kinetic assay because it inhibits the early steps of glycolysis (hexokinase) (21) and subsequently downstream reactions, including the pyrophosphate pathway and the tricarboxylic acid cycle. We employed diamide, an established thiol-oxidizing agent that oxidizes GSH to GS-SG (22), to induce GSR-mediated NADPH consumption during reduction of GS-SG back to GSH (Fig 1A). By adding diamide and 2-DG simultaneously to LPS-stimulated cultured Mφs and measuring NADPH over a defined time interval, we can assess NADPH consumption by GSR in a kinetic assay.

As was described above, GSR is localized in different subcellular compartments and may differentially regulate NADPH levels. Conventional commercial kits that measure NADPH cannot provide organelle-specific information, which is an important limitation. To address this, we integrated organelle specific $NADP^+$ sensors into our assay. The Apollo-$NADP^+$ sensors are fluorescently tagged enzymatically inactive G6PD constructs that enable NADPH/$NADP^+$ redox states measurement through fluorescence anisotropy (Fig 1B) (23). Recently, Chang et al improved the sensors by adding sequences that target protein expression to specific organelles, such as mitochondria (24). Therefore, by transfecting Mφ cell lines with targeted Apollo-$NADP^+$ sensors and treating cells with 2-DG and diamide, we can measure NADPH consumption by GSR in the cytosol or mitochondria in a kinetic assay.

Materials

Biological materials:

1. RAW264.7 Mφ cell line (ATCC, Catalog #:TIB-71)

Reagents:

1. 0.25% Trypsin/ 0.1% EDTA (Wisent, Catalog#:325-043 EL)
2. DMEM 1X with 4.5 g/L Glucose, L-Glutamine & Sodium Pyruvate (Wisent, Catalog#:319-005-CL)
3. Premium Fetal Bovie Serum (FBS) Endotoxin < 1 EU/ml (Wisent, Catalog#:091-150)
4. PBS 1X, w/o Calcium & Magnesium (Wisent, Catalog#:311-010 CL)
5. Geneticin™ Selective Antibiotic (G418 Sulfate) (50 mg/mL) (ThermoFisher, Catalog#: 10131035)
6. Amaxa® Cell Line Nucleofector® Kit V (LONZA, Catalog#: VCA-1003)
7. HBSS, no calcium, no magnesium, no phenol red (HBSS) 1X (ThermoFisher, Catalog#: 14175095)
8. Diamide (Sigma Aldrich, Catalog#: D3648)
9. 2-DG (Sigma Aldrich, Catalog#: D8375)
10. Mitochondrial mTurq2-Apollo-NADP+ (Addgene, Catalog#: 193303)
11. Cytosolic mVenus-Apollo-NADP+ (Addgene, Catalog#: 71797)
12. Fluorescein (Sigma Aldrich, Catalog#: 46955)

13. BMHH buffer (125 mm NaCl, 5.7 mm KCl, 2.5 mm CaCl2, 1.2 mm MgCl2, 10 mm HEPES, and 0.1% bovine serum albumin, pH 7.4)
14. Trypan Blue Solution (Sigma, Catalog#: T8154)

Solutions and Recipes:

1. BMHH imaging buffer (125 mM NaCl, 5.7 mM KCl, 2.5 mM CaCl2, 1.2 mM MgCl2, 0.1% bovine serum albumin (BSA), and 10 mM 4-(2-hydroxyethyl)-1- piperazineethanesulfonic acid (HEPES) at pH 7.4).

Laboratory supplies and equipment:

1. Hemocytometer cell counting chamber (Sigma, Catalog#: BR719505-1EA)
2. Lonza™ Nucleofector™ Transfection 2b Device (Fisher Scientific, Catalog#: 13458999)
3. T75 tissue culture treated flask (StemCell Technologies, Catalog#: 200-0501)
4. T25 tissue culture treated flask (Sigma, Catalog#: C6356-200EA)
5. 35 mm Dish (No. 1.5 Coverslip, 14 mm Glass Diameter, Uncoated) (Mattek, Catalog#: P35G-1.5-14-C)
6. Custom-built wide-field RAMM microscope (ASI) equipped with excitation light-emitting diodes (LEDs) (405, 505, and 590 nm) and an excitation polarizing filter (Edmund Optics, Barrington, NJ), and built to perform simultaneous two-color fluorescence anisotropy imaging.
7. Images were collected using 40×/0.75 NA air objective lens (Olympus, Richmond Hill, Canada).
8. Fluorescence was passed through a Cerulean (ET470/24M) or Venus (ET535/30M) emission filter on a filter wheel, and subsequently split using an Optosplit II (Cairn, Faversham, U.K.) to simultaneously collect parallel and perpendicular emission light on separate regions of an IRIS 15 CMOS camera (Teledyne Photometrics, Tucson, AZ). Excitation was also passed through a triple band filter containing ET-350/55-470/30-557/35 band-pass filters.

Procedures

Part I: Transfection of Apollo-$NADP^+$ sensors in RAW264.7 Mφ cell lines

1. One week prior to nucleofection, passage cells in 15mL of DMEM media supplemented with 10% FBS for every 2-3 days in a T75 tissue culture treated flask.
2. To passage cells, remove cultured media and treat cells with 10mL of trypsin for 5 minutes at 37°C/5% CO_2 for detachment. Next, add 10mL of fresh DMEM media to neutralize trypsin and centrifuge the cells at 90xg for 5 minutes.
3. Remove the supernatant, resuspend the cells with 10mL of fresh DMEM media, quantify the number of cells using a hemocytometer cell counting chamber and passage them at a ratio of 1:5 to 1:8.
4. One day prior to nucleofection, passage the cells at a ratio of 1:3.
5. On the day of nucleofection, retrieve the nucleofector solution and supplement from Amaxa® Cell Line Nucleofector® Kit V. For the first time usage, ensure to add all the supplement to the solution

6. Fill one well of a 6-well plate with 1 mL of DMEM that is supplemented with 20% FBS. Pre-warm the plate at 37°C/5% CO_2.
7. Centrifuge and isolate 2 x 10^6 cells. Remove supernatant and rinse cells with 1mL of pre-warmed 1X PBS. Repeat centrifugation, remove PBS and resuspend pellet with 100 µl of supplemented nucleofector solution. Add 5 µg of mitochondrial mTurq2-Apollo-$NADP^+$ sensor or cytosolic mVenus-Apollo-$NADP^+$ sensor to the mixture.
8. Transfer the complete mixture to kit-supplied cuvette without the injection of bubbles. Insert the cuvette into a Nucleofector® II Device and select program D-032 by pressing the X-button. Upon completion of nucleofection, retrieve the cuvette and add 500 µl of pre-warmed DMEM supplemented with 20% FBS into the suspension.
9. Transfer the entire 600ul of suspension using kit-supplied pipettes into the pre-warmed 6 well-plate in a drop-wise manner (total volume is 1.6 mL in the well).
10. 3 hours after the cells have adhered, replace the cultured media with 2mL of fresh DMEM media supplemented with 20% FBS for recovery.
11. When cell confluency has reached a minimum of 80% (usually 48 hours post transfection), the cells can be replated for experiments where transient nucleofection of the sensor is desired.
12. For stable selection, re-seed all the transfected cells from step 11 to a T25 culture flask with 5mL of DMEM media supplemented with 10% FBS. 3 hours after the cells have adhered, replace the cultured media with fresh DMEM media supplemented with 10% FBS and G418 Sulfate at final concentration of 400 µg/ml.
13. Replace the media with fresh 5mL of DMEM media supplemented with 10% FBS and G418 for every 2-3 days until one week. If cell confluency has reached a minimum of 80% in the T25 flask, re-seed the cells to a T75 flask or 10 cm tissue culture treated dish with 10mL of DMEM media supplemented with 10% FBS and G418.
14. To set up imaging of the sensors, isolate and seed 1x10^6 cells in a series of 35-mm petri dish (one dish per time point of the kinetic) with 2mL of DMEM media supplemented with 10% FBS and G418 one day prior to imaging.

Part II: Imaging of the Apollo-$NADP^+$ sensors in RAW264.7 Mφ cell lines

1. On the day of imaging, prepare a 1:1 mixture of diamide (250 mM) and 2-DG (250 mM) in HBSS buffer. Specifically, add 1mL of 500 mM of diamide with 1mL of 500 mM of 2-DG.
2. On the day of imaging, prepare and calibrate microscope for imaging of Apollo-NADP+ sensor.
    a. Turn on microscope at least 30min prior to any imaging to ensure CMOS camera temperature reaches equilibrium (Iris 15 camera was used in this case).
    b. Launch microscope management software, in this case open source software Micro-Manage was used (available for download at https://micro-manager.org/Download_Micro-Manager_Latest_Release)
        i. Set binning. In this case binning was set to 4x4 to collect the maximum image intensity.
        ii. Live imaging parameters should be set low to limit photobleaching and phototoxicity. In this case LED and exposure time were set to 20% and 100 ms, respectively.
    c. Ensure LED is properly aligned (i.e., evenly illuminates the back aperture of the lens) by first turning on the YFP LED, then holding a piece of paper against the

microscope stage adjust the LED position until the emitted light appearing on the paper becomes sharp.

   d. Calibrate polarizer by first ensuring the polarizer is placed in the excitation path.
      i. Next, place a YFP-autofluorescent-plastic-slide on the microscope stage.
      ii. Begin live imaging at low intensity (20%) and low exposure time (10-50 ms), then using the draw tool draw a box around the live image of both the parallel and perpendicular channels.
      iii. Then select 'Analyze' > 'Plot Profile'. This will display a live profile of the intensity of both the parallel and perpendicular channels.
      iv. Finally, adjust the polarizer until the difference in intensity values between the parallel and perpendiculr channels is maximized. When done correctly the parallel channel will have an overall higher intensity than perpendicular.

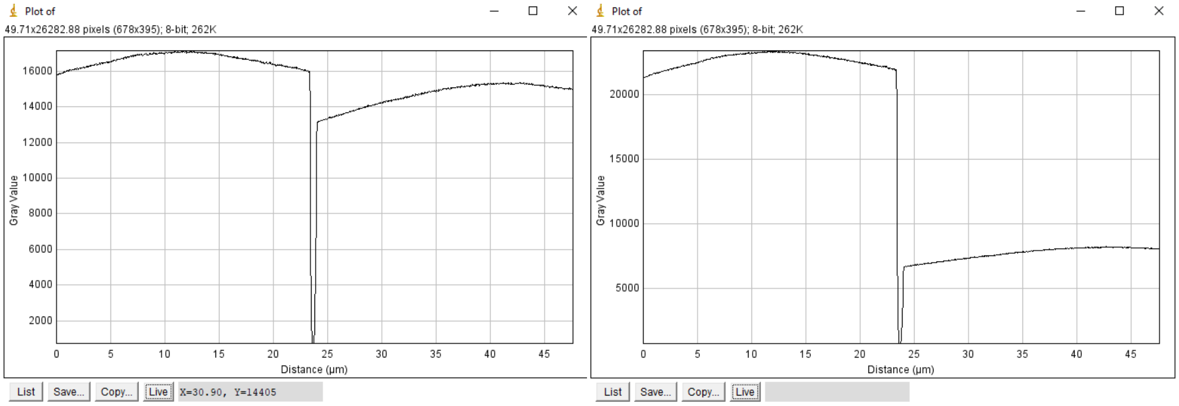

Figure 1: For step d,iv. Left: incorrectly adjusted polarizer. Right: Correctly adjusted polarizer

3. Remove the cultured media and replace it with 900µl of pre-warmed HBSS.
4. Determine image acquisition parameters, LED intensity (%) and exposure time (ms).
   a. Place cells in pre-warmed HBSS onto stage top and use brightfield settings locate and focus on the cells.
   b. Switch to fluorescence imagine (e.g., YFP imaging), and use live imaging settings to fine focus on the cells/sensor. Set LED intensity to 80% and experiment with different image acquisition exposure times to find parameters that produce final sensor intensities within your camera's known saturation levels. In this case the total intensity in the parallel channel was roughly 40,000 AU, which was commonly achieved with an LED intensity of 80% and exposure time of 800 ms.
5. For the first time point, do not add any diamide and 2-DG. Image the sensor to acquire the baseline value of the control and experimental condition.
   a. TROUBLESHOOTING: Please refer to *General Notes and Troubleshooting* if the sensors were not expressed or weakly expressed.
6. For the second time point, add 100µl of the 1:1 mixture of diamide and 2-DG into the first petri dish, such that the final concentration of diamide and 2-DG is 25mM respectively. Incubate for 5 minutes, followed by imaging of the sensors.

7. For the third and subsequent time points, repeat step 6 for an additional 5 minute-interval until the completion of the kinetics.
8. At the end of imaging acquire images of standard anisotropy fluorescein solution for instrument diagnostics and G-factor correction.
    a. Start by preparing a solution of 5 mM fluorescein dissolved in BMHH.
    b. Set LED intensity and exposure time such that the acquired image has roughly the same total intensity of the sensor in cells, in this case 40,000 AU at 20% LED and 20 ms exposure time.
    c. Fluorescein solution can be left in the fridge at 4°C for 1 month for repeated uses.

Part IV: Data analysis

1. Parallel ($I_\parallel$) and perpendicular ($I_\perp$) fluorescence intensity images were analyzed with a custom ImageJ plugin. These are available at https://github.com/RocheleauLab/Optosplit-Anisotropy-Analysis-scripts. The following three imageJ macro scripts are required:
    a. 00-Preporcessing
    b. 01-Anisotropy Macro
    c. 03-ROI Selection
2. The images were background-corrected using a rolling ball filter where the radius is larger than the largest cell in your images but small enough that distortions in the background are removed, in this case a radius of 100 was used. Pixel-by-pixel anisotropy (r) was calculated using the background-corrected intensities: $r = (I_\parallel - GI_\perp)/(I_\parallel + 2GI_\perp)$.
    a. Start by navigating to the folder containing all images and create the following two folders:
        i. GfactorImages
        ii. AlignmentImages
    b. Drag and drop your fluorescein image into the GfactorImages folder.
    c. Select an image of your cells and drag and drop into the imageJ application.
        i. On the imageJ application select Plugins > Macros > Record…
        ii. Using the draw Rectangle tool, draw two boxes of the same size and position on the parallel and perpendicular channels of the image

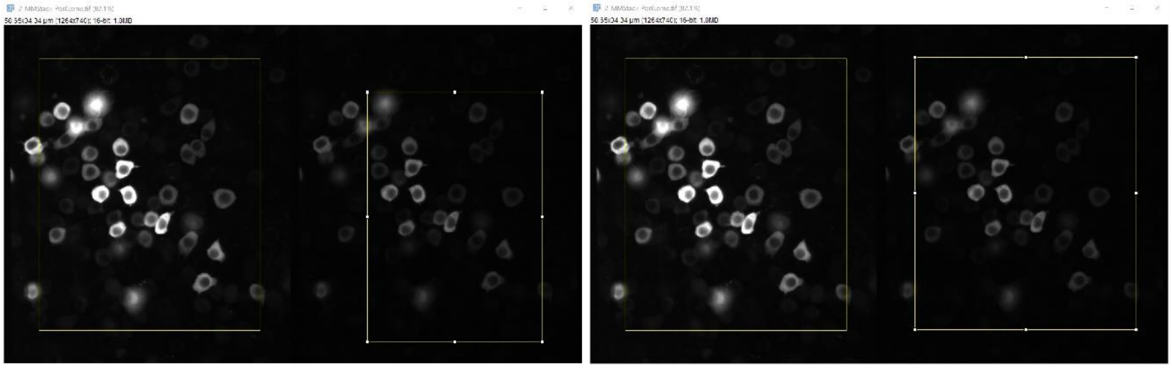

Figure 2: For step c,ii. Left: incorrectly drawn boxes, right: correctly drawn boxes

   iii. Dimensions for the boxes drawn will appear in the Record dialogue box. Copy and paste these dimensions into 00-Preprocessing script under rect_para and rect_perp
   iv. Run 00-Preprocessing script
   v. A file explore dialogue box will open. Navigate to Alignment Folder > Transform "select"
   vi. Another file explore dialogue box will open. This time navigate to Alignment Folder > Original > and "select" the image titled "Para"
   vii. The script will continue running through all the images
   viii. Processed images will be placed in a new folder titled "T2_Processed"
   ix. The script will continue running through all images in the experiment folder. This may take several minutes and depends on the number of images acquired

3. Gfactor for the wide-field microscope was calculated using fluorescein solutions, which have an anisotropy near zero, thus simplifying the standard anisotropy equation to $G = I_{\parallel}/I_{\perp}$.
   a. After 00-Preprocessing script has finished running, navigate to 01-Anisotropy Macro script and press Run
   b. A file explorer dialogue box will open, select the experiment folder you are working with
   c. Allow the script to run. This may take several minutes depending on the number of images acquired
4. Regions of interest (ROI) were selected using the parallel intensity images to avoid selection bias.
   a. After 01-Anisotropy Macro script has finished, navigate to 03-ROI Selection and press Run
   b. Using either the oval tool or the 'any shape tool' draw regions of interest over the largest portion of where the sensor is appearing in the Para channel
5. Each independent trial consists of at least five to more than 30 cells (5−30 technical replicates).

Other procedures are described below.

Immunoblotting

RAW264.7 cells were cultured in 12-well plates at 2 x 10^6 cells per well/experimental condition. Cells were lysed for 15 minutes in ice-cold RIPA buffer consisting of 1% NP40, 0.1% SDS, 0.5% deoxycholate in PBS, supplemented with 1 mM PMSF, 1X cOmplete™, EDTA-free Mini Protease Inhibitor Cocktail (Sigma Cat#11873580001) and 1X PhosSTOP™ (Sigma Cat#4906845001). Protein concentrations in lysates were determined by Protein Assay Dye Reagent (BioRad #5000006), diluted in 2x Laemmli sample buffer (BioRad Cat#161-0737) with fresh β-mercaptoethanol (BioRad #1610710), and heated at 95°C for 5 minutes. Samples (20 µg of protein per lane) were resolved on 8% SDS-PAGE gels and transferred to polyvinylidene difluoride membranes (Sigma #IPVH00010) using a wet transfer system. Membranes were blocked with 5% skim milk non-fat powder in Tris-buffered saline-Tween for 1 h at room temperature. Membranes were incubated with primary antibodies overnight: anti-Actin (Sigma, A2066), anti-NRF2 (Cell Signaling Technology #12721) and anti-GSR (ThermoFisher, PA5-29945), followed by washing and incubation with HRP-conjugated anti-rabbit IgG (CST#7074) (22°C, 1 h). Blots were developed using Immobilon Forte Western HRP substrate (Sigma, WBLUF0100) and imaged with Microchemi 4.2 (BioRad).

Quantification of total NADPH levels

Total NADPH assay kits (Abcam, #ab186031) were used. In brief, cells were cultured in 6-well plates, then loaded with oxLDL for 24 h with subsequent 6 h LPS stimulation the next day. Cells were then lysed with respective lysis buffers provided in the kits and the abundance of metabolites was determined according to the manufacturers' protocols.

RNA isolation and real-time quantitative PCR

Total RNA was isolated with E.Z.N.A.® Total RNA Kit I (Omega Cat#R6834-01) and reverse transcription (RT) reactions were performed with High-Capacity cDNA Reverse Transcription Kit (ThermoFisher Cat#4368814) according to manufacturer's protocol. Real-time quantitative-PCR (qPCR) was then performed using Roche LightCycler 480 with Luna® Universal qPCR Master Mix (New England Biolabs, Cat#M3003E). Quantification of mRNA was performed by using primers that span over two adjacent exons, quantified using the comparative standard curve method and normalized to hypoxanthine phosphoribosyltransferase (*Hprt*), a housekeeping gene. Primer sequences used for qPCR are listed below:

Gsr Fwd: GTTTACCGCTCCACACATCCTG Rev: GCTGAAAGAAGCCATCACTGGTG

Gclm Fwd: TCCTGCTGTGTGATGCCACCAG Rev: GCTTCCTGGAAACTTGCCTCAG

Nqo1 Fwd: GCCGAACACAAGAAGCTGGAAG Rev: GCAAATCCTGCTACGAGCACT

Txnrd1 Fwd: AGTCACATCGGCTCGCTGAACT Rev: GATGAGGAACCGCTCTGCTGAA

Srxn1 Fwd: TACCAATCGCCGTGCTCATCCG Rev: CCTTTGATCCAGAGGACGTCGA

Hmox1 Fwd: CACTCTGGAGATGACACCTGAG Rev: GTGTTCCTCTGTCAGCATCACC

Hprt Fwd: CAAGCTTGCTGGTGAAAAGGA Rev: TGAAGTACTCATTATAGTCAAGGGCATATC

General Notes and Troubleshooting

Although the expression levels of cytosolic and mitochondrial Apollo-NADP$^+$ sensors were adequate in transiently (>48 h) and stably (>1 week) transfected RAW264.7 cells, we found the quantification of anisotropy to be less variable in stably transfected cells. Therefore, we recommend the use of stably transfected cells for experimental data acquisition, while transiently transfected cells can be used to verify targeting of specific organelles by different sensors.

Since the Apollo-NADP$^+$ sensor response depends on the interaction (dimerization) of exogenous mutant G6PD tagged with a fluorescent protein, interactions between transfected and endogenous G6PD may reduce sensor response. Therefore, in cell types with large amounts of endogenous G6PD, the apparent dynamic range of the sensor's response may be reduced. To confirm that the sensor responds in a particular cell type, diamide can be used to induce sensor dimerization and the magnitude of the response can be measured.

Step	Problem	Possible reason	Solution
Imaging of cytosolic and/or mitochondrial Apollo-NADP$^+$ sensor post transient nucleofection.	No or weak expression of cytosolic and mitochondrial sensor post transient nucleofection.	Insufficient amount of plasmid was added during nucleofection.	1. Increase the amount of plasmid (up to 5µg) per nucleofection.<br>2. Ensure the plasmid was properly extracted during plasmid preparation with no LPS contamination.<br>3. Sequence the plasmid to ensure the sequence matches with the original listed sequence posted on Addgene.
		Incorrect time-window to detect the expression of plasmid.	Determine the best time-window to detect the expression of plasmid from 24h to 72h post transient nucleofection.
Imaging of cytosolic and/or mitochondrial Apollo-NADP$^+$ sensor post stable nucleofection.	No or weak expression of cytosolic and mitochondrial sensor post transient nucleofection.	Incorrect dosage of G418 was used to stably select transfected cells.	Perform a serial-dilution of G418 and determine the minimum concentration needed to kill non-transfected cells within one week.

		The weakly expressed clones were outcompeting the strongly expressed clones.	Perform clonal selection and expansion post stable nucleofection. Characterize all the isolated clones and select the strongly expressed ones for future experiments.
Drawing regions of interest (ROI) during analysis.	Anisotropy values vary with certain regions of the cell.	Cell nucleus excludes the sensor and therefore does not exhibit valid anisotropy values.	When drawing the ROI's ensure that the region is within the cell boundary and does not include the nucleus.
		Edges of the cell express unreliable anisotropy values	

Expected Results

Prior to the validation of our assay, we increased the expression of GSR by loading RAW264.7 Mφs with oxidized low-density lipoprotein (oxLDL) and then stimulating with LPS. This was based on our previous reports that incubating primary murine Mφs with oxLDL or cholesterol is a reproducible method of lipid loading Mφs, and this significantly upregulated NRF2-dependent transcriptional regulation after LPS stimulation (6, 25, 26). These experimental conditions are important as increasing GSR protein expression will directly increase its activity and consumption of NADPH, thereby serving as a positive control for our assay. GSR is a NRF2-regulated gene (27). Therefore, as expected we found increased mRNA expression of *Gsr* and other NRF2-regulated genes (*Gclm*, *Nqo1*, *Srxn1* and *Hmox1*) (Fig 2A), as well as NRF2 and GSR protein abundance (Fig 2B) in LPS-stimulated RAW264.7 Mφs with accumulated oxLDL. These data confirm that oxLDL loading of RAW264.7 Mφs increases GSR abundance after LPS stimulation and thus justify the use of these conditions to validate our proposed assay.

We measured the rate of NADPH consumption by GSR in whole cells using a commercial kit that quantifies NADPH. The concentration of NADPH in a cell lysate is determined by the colorimetric absorbance after adding the supplied NADPH probe solution. After confirming that the concentration of NADPH in our lysates was within the linear detection range of the assay (Fig 3A), we treated LPS-stimulated (6 h) RAW264.7 Mφs with 2-DG and diamide and quantified NADPH kinetics by obtaining lysates at 5-minute intervals for a total of 25 minutes (Fig 3B). Whole cell NADPH was reduced in a time-dependent manner, suggesting that NADPH was being consumed by GSR. Notably, in oxLDL loaded Mφs, NADPH was reduced more rapidly. This is expected because GSR mRNA and protein expression is higher in these Mφs (Fig 2).

After confirming the feasibility of our proposed kinetic assay, we proceeded to integrate determination of NADPH in subcellular compartments using targeted Apollo-$NADP^+$ sensors. To do this, we first confirmed that both cytosolic- and mitochondrial-targeted Apollo-$NADP^+$ sensors are expressed in appropriate compartments of RAW264.7 Mφs. Transient transfection of

cytosolic and mitochondrial Apollo-$NADP^+$ sensors was detected by fluorescence microscopy 48 h post-nucleofection (Fig 4A). One week of geneticin selection post-nucleofection resulted in stable expression of both sensors (Fig 4B). To test the functionality of the sensors, we cultured transiently-transfected RAW 264.7 Mφs with or without oxLDL for 24 h, stimulated the cells with LPS for 6 h and performed imaging of the sensors. OxLDL loading significantly inhibited anisotropy in both the cytosol and mitochondrial matrix (Fig 4C), which indicates a higher $NADP^+$ to NADPH ratio. Finally, to determine if the reduction of NADPH to $NADP^+$ was due to increased consumption by GSR, we performed a kinetic assay using stably-transfected RAW 264.7 Mφs with or without oxLDL loading, LPS stimulation and the addition of 2-DG and diamide. OxLDL loading significantly depleted cytosolic and mitochondrial anisotropy in a time-dependent manner (Fig 4D), suggesting that oxLDL loading increased GSR-mediated NADPH consumption in both compartments.

List of Abbreviations

Abbreviations	Definition
$NADP^+$	Nicotinamide adenine dinucleotide phosphate
GSR	Glutathione-disulfide reductase
GS-SG	Glutathione disulfide
GSH	Glutathione
ROS	Reactive oxygen species
LPS	Lipopolysaccharide
G6PD	Glucose-6-phosphate dehydrogenase
2-DG	2-deoxyglucose
oxLDL	Oxidized low-density lipoprotein
NRF2	Nuclear factor erythroid 2-related factor 2

Declarations

Ethics approval and consent to participate

Not applicable

Consent for publication

Not applicable

Availability of data and materials

All data generated or analyzed during this study are included in this published article [and its supplementary information files].

Competing interests

The authors declare that they have no competing interests.

Funding

This work was supported by the Canadian Institutes of Health Research Grant FDN154299 (to M.I.C.). Additional support of this work was from Canadian Institutes of Health Research Project Grant DOL 409157 (to J.R.), and by Natural Sciences and Engineering Research Council of Canada Research Tools and Instrumentation Grant 2018-00846 (to J.R.). M.I.C. holds a Tier 1

Canada Research Chair. K.K.Y.T. was supported by fellowships from the Canadian Institutes of Health Research, Ontario Graduate Scholarship, fellowships from University of Toronto and The Peterborough K. M. Hunter Charitable Foundation.

Authors' contributions

Conceptualization, K.K.Y.T., E.F.; methods, K.K.Y.T., E.F.; software, E.F.; investigation, K.K.Y.T., E.F., R.D.; formal analysis, K.K.Y.T., E.F.; resources, J.R.; visualization, K.K.Y.T. E.F., E.F.; writing— original draft, K.K.Y.T., E.F.; writing—review and editing, K.K.Y.T., E.F., J.R., M.I.C; supervision, J.R., J.J-B., M.I.C.; funding acquisition, K.K.Y.T., M.I.C., J.R.

Acknowledgements

Not applicable

References

1. Ting KKY, Jongstra-Bilen J, Cybulsky MI. 2023. The multi-faceted role of NADPH in regulating inflammation in activated myeloid cells. *Front Immunol* 14: 1328484
2. Everts B, Amiel E, Huang SC, Smith AM, Chang CH, Lam WY, Redmann V, Freitas TC, Blagih J, van der Windt GJ, Artyomov MN, Jones RG, Pearce EL, Pearce EJ. 2014. TLR-driven early glycolytic reprogramming via the kinases TBK1-IKKε supports the anabolic demands of dendritic cell activation. *Nat Immunol* 15: 323-32
3. Fresta CG, Fidilio A, Lazzarino G, Musso N, Grasso M, Merlo S, Amorini AM, Bucolo C, Tavazzi B, Lazzarino G, Lunte SM, Caraci F, Caruso G. 2020. Modulation of Pro-Oxidant and Pro-Inflammatory Activities of M1 Macrophages by the Natural Dipeptide Carnosine. *Int J Mol Sci* 21
4. Britt EC, Lika J, Giese MA, Schoen TJ, Seim GL, Huang Z, Lee PY, Huttenlocher A, Fan J. 2022. Switching to the cyclic pentose phosphate pathway powers the oxidative burst in activated neutrophils. *Nat Metab* 4: 389-403
5. Noda T, Amano F. 1997. Differences in nitric oxide synthase activity in a macrophage-like cell line, RAW264.7 cells, treated with lipopolysaccharide (LPS) in the presence or absence of interferon-gamma (IFN-gamma): possible heterogeneity of iNOS activity. *J Biochem* 121: 38-46
6. Ting KKY, Yu P, Dow R, Floro E, Ibrahim H, Scipione CA, Hyduk SJ, Polenz CK, Zaslaver O, Karmaus PWF, Fessler MB, Röst HL, Ohh M, Tsai S, Winer DA, Woo M, Rocheleau J, Jongstra-Bilen J, Cybulsky MI. 2023. Oxidized Low-Density Lipoprotein Accumulation Suppresses Glycolysis and Attenuates the Macrophage Inflammatory Response by Diverting Transcription from the HIF-1α to the Nrf2 Pathway. *J Immunol* 211: 1561-77
7. Fan J, Ye J, Kamphorst JJ, Shlomi T, Thompson CB, Rabinowitz JD. 2014. Quantitative flux analysis reveals folate-dependent NADPH production. *Nature* 510: 298-302
8. Dayaram YK, Talaue MT, Connell ND, Venketaraman V. 2006. Characterization of a glutathione metabolic mutant of Mycobacterium tuberculosis and its resistance to glutathione and nitrosoglutathione. *Journal of Bacteriology* 188: 1364-72
9. Ricci V, Giannouli M, Romano M, Zarrilli R. 2014. Helicobacter pylori gamma-glutamyl transpeptidase and its pathogenic role. *World Journal of Gastroenterology* 20: 630-8
10. Ghosh A, Raha S. 2015. Molecular and functional characterisation of a stress responsive cysteine protease, EhCP6 from Entamoeba histolytica. *Protein Expression and Purification* 109: 55-61

11. Raj D, Ghosh E, Mukherjee AK, Nozaki T, Ganguly S. 2014. Differential gene expression in giardia lamblia under oxidative stress: Significance in eukaryotic evolution. *Gene* 535: 131-9
12. Couto N, Wood J, Barber J. 2016. The role of glutathione reductase and related enzymes on cellular redox homoeostasis network. *Free Radical Biology and Medicine* 95: 27-42
13. Oliveira P, Martins NM, Santos M, Couto NAS, Wright PC, Tamagnini P. 2015. The anabaena sp. PCC 7120 exoproteome: Taking a peek outside the box. *Life* 5: 130-63
14. Couto N, Schooling SR, Dutcher JR, Barber J. 2015. Proteome Profiles of Outer Membrane Vesicles and Extracellular Matrix of Pseudomonas aeruginosa Biofilms. *Journal of Proteome Research* 14: 4207-22
15. Outten CE, Culotta VC. 2004. Alternative Start Sites in the Saccharomyces cerevisiae GLR1 Gene Are Responsible for Mitochondrial and Cytosolic Isoforms of Glutathione Reductase. *Journal of Biological Chemistry* 279: 7785-91
16. Couto N, Malys N, Gaskell SJ, Barber J. 2013. Partition and turnover of glutathione reductase from saccharomyces cerevisiae: A proteomic approach. *Journal of Proteome Research* 12: 2885-94
17. Ge C, Spånning E, Glaser E, Wieslander A. 2014. Import determinants of organelle-specific and dual targeting peptides of mitochondria and chloroplasts in arabidopsis thaliana. *Molecular Plant* 7: 121-36
18. Ham M, Lee JW, Choi AH, Jang H, Choi G, Park J, Kozuka C, Sears DD, Masuzaki H, Kim JB. 2013. Macrophage glucose-6-phosphate dehydrogenase stimulates proinflammatory responses with oxidative stress. *Mol Cell Biol* 33: 2425-35
19. Maeng O, Kim YC, Shin HJ, Lee JO, Huh TL, Kang KI, Kim YS, Paik SG, Lee H. 2004. Cytosolic NADP(+)-dependent isocitrate dehydrogenase protects macrophages from LPS-induced nitric oxide and reactive oxygen species. *Biochem Biophys Res Commun* 317: 558-64
20. Yu W, Wang Z, Zhang K, Chi Z, Xu T, Jiang D, Chen S, Li W, Yang X, Zhang X, Wu Y, Wang D. 2019. One-Carbon Metabolism Supports S-Adenosylmethionine and Histone Methylation to Drive Inflammatory Macrophages. *Mol Cell* 75: 1147-60.e5
21. Pajak B, Siwiak E, Sołtyka M, Priebe A, Zieliński R, Fokt I, Ziemniak M, Jaśkiewicz A, Borowski R, Domoradzki T, Priebe W. 2019. 2-Deoxy-d-Glucose and Its Analogs: From Diagnostic to Therapeutic Agents. *Int J Mol Sci* 21
22. Kosower NS, Kosower EM, Wertheim B, Correa WS. 1969. Diamide, a new reagent for the intracellular oxidation of glutathione to the disulfide. *Biochemical and Biophysical Research Communications* 37: 593-6
23. Cameron WD, Bui CV, Hutchinson A, Loppnau P, Gräslund S, Rocheleau JV. 2016. Apollo-NADP+: a spectrally tunable family of genetically encoded sensors for NADP+. *Nature Methods* 13: 352-8
24. Chang HH, Bennett AM, Cameron WD, Floro E, Au A, McFaul CM, Yip CM, Rocheleau JV. 2022. Targeting Apollo-NADP(+) to Image NADPH Generation in Pancreatic Beta-Cell Organelles. *ACS Sens* 7: 3308-17
25. Ting KKY YP, Dow R, Ibrahim H, Karim S, Polenz CK, Winer DA, Woo M, Jongstra-Bilen J, Cybulsky MI. Cholesterol accumulation impairs HIF-1α-dependent immunometabolic reprogramming of LPS-stimulated macrophages by upregulating the NRF2 pathway. Sci Rep. 2024. (In press)
26. Ting KKY, Yu P, Iyayi M, Dow R, Hyduk SJ, Floro E, et al. Oxidized Low-Density Lipoprotein Accumulation in Macrophages Impairs Lipopolysaccharide-Induced Activation of AKT2, ATP Citrate Lyase, Acetyl-Coenzyme A Production, and Inflammatory Gene H3K27 Acetylation. Immunohorizons. 2024;8(1):57-73.

27. Harvey CJ, Thimmulappa RK, Singh A, Blake DJ, Ling G, Wakabayashi N, Fujii J, Myers A, Biswal S. 2009. Nrf2-regulated glutathione recycling independent of biosynthesis is critical for cell survival during oxidative stress. *Free Radic Biol Med* 46: 443-53

Figure Legends

Figure 1. Schematic of the assay. (A) The entire workflow is illustrated. The assay begins with nucleofection of cytosolic mVenus-Apollo-$NADP^+$ and mitochondrial mTurq2-Apollo-$NADP^+$ sensors into RAW264.7 Mφ cell line, followed by imaging of the sensors. In a kinetic assay, 2-deoxyglucose (2-DG) and diamide are added together prior to an imaging time series. The addition of 2-DG blocks biochemical pathways that reduce $NADP^+$ to NADPH in LPS-stimulated cells. The addition of diamide oxidizes GSH to GS-SG, thereby stimulating the activity of GSR to reduce GS-SG back to GSH in a NADPH-dependent manner. **(B)** The mechanism of the Apollo-$NADP^+$ sensor is illustrated. The sensor, which is based on an enzymatically inactivated human G6PD tagged with a fluorescent protein, undergoes homodimerization in response to changes in free $NADP^+$ levels. This $NADP^+$-dependent dimerization/monomerization modulates FRET between the homologous fluorescent proteins (homoFRET) resulting in changes in steady-state fluorescence anisotropy that can then be used to assess the redox state of $NADP^+$/NADPH. (Created with Biorender.com).

Figure 2. OxLDL loading of RAW264.7 Mφs upregulates NRF2-dependent transcriptional regulation post LPS stimulation. (A) qPCR data showing the normalized mRNA expression of NRF2-regulated genes (*Gclm, Nqo1, Srxn1, Hmox1, Gsr*) in RAW264.7 Mφs with (+) and without (-) oxLDL loading and LPS stimulation for 6 h (n=4-5). Values are normalized to the group without oxLDL loading. The mean ± SEM is plotted. Significant differences were determined by an unpaired Student's *t*-test (*P<0.05, **P<0.01, ***P<0.001). **(B)** Representative immunoblots showing NRF2, GSR and actin protein expression in RAW264.7Mφs with and without oxLDL loading. A LPS stimulation time course (0 to 6 h) was performed.

Figure 3. OxLDL loading of RAW264.7 Mφs enhances GSR-dependent NADPH consumption 6 hours after LPS stimulation. (A) Representative NADPH standard curve generated for analysis of RAW264.7 Mφs-derived lysates. **(B)** Representative experiment showing the quantification of NADPH abundance in RAW264.7Mφs with (+) and without (-) oxLDL loading and 6 h post-LPS stimulation. Samples were obtained at sequential time points after the addition of 2-deoxyglucose (2-DG) and diamide.

Figure 4. OxLDL loading of RAW264.7 Mφs enhances GSR-dependent NADPH consumption in the mitochondria and the cytosol 6 hours after LPS stimulation. (A, B) Representative microscopic images of cytosolic mVenus-Apollo-$NADP^+$ and mitochondrial mTurq2-Apollo-$NADP^+$ sensors 48 h after transient nucleofection (A) and 1 week after stable transfection (B). Magnification bars represent 20 µm. **(C)** 1P anisotropy quantification of cytosolic mVenus-Apollo-$NADP^+$ and mitochondrial mTurq2-Apollo-$NADP^+$ sensors in transiently-transfected RAW264.7 cells with (+) and without (-) oxLDL loading and 6 h of LPS stimulation (n=3). **(D)** Representative kinetic assay showing quantification of 1P anisotropy of cytosolic mVenus-Apollo-$NADP^+$ and mitochondrial mTurq2-Apollo-$NADP^+$ sensors in stably-transfected RAW264.7 cells ±oxLDL loading, LPS stimulation (6 h) and treatment with 2-DG and diamide. The mean ± SEM is plotted in all graphs. Significant differences (*P<0.05, **P<0.01,

****P<0.0001) were determined by an unpaired Student's *t*-test in (C) or by a two-way ANOVA with Bonferroni correction in (D).

Click here to access/download;Figure;Figure_1.pdf

Figure 1

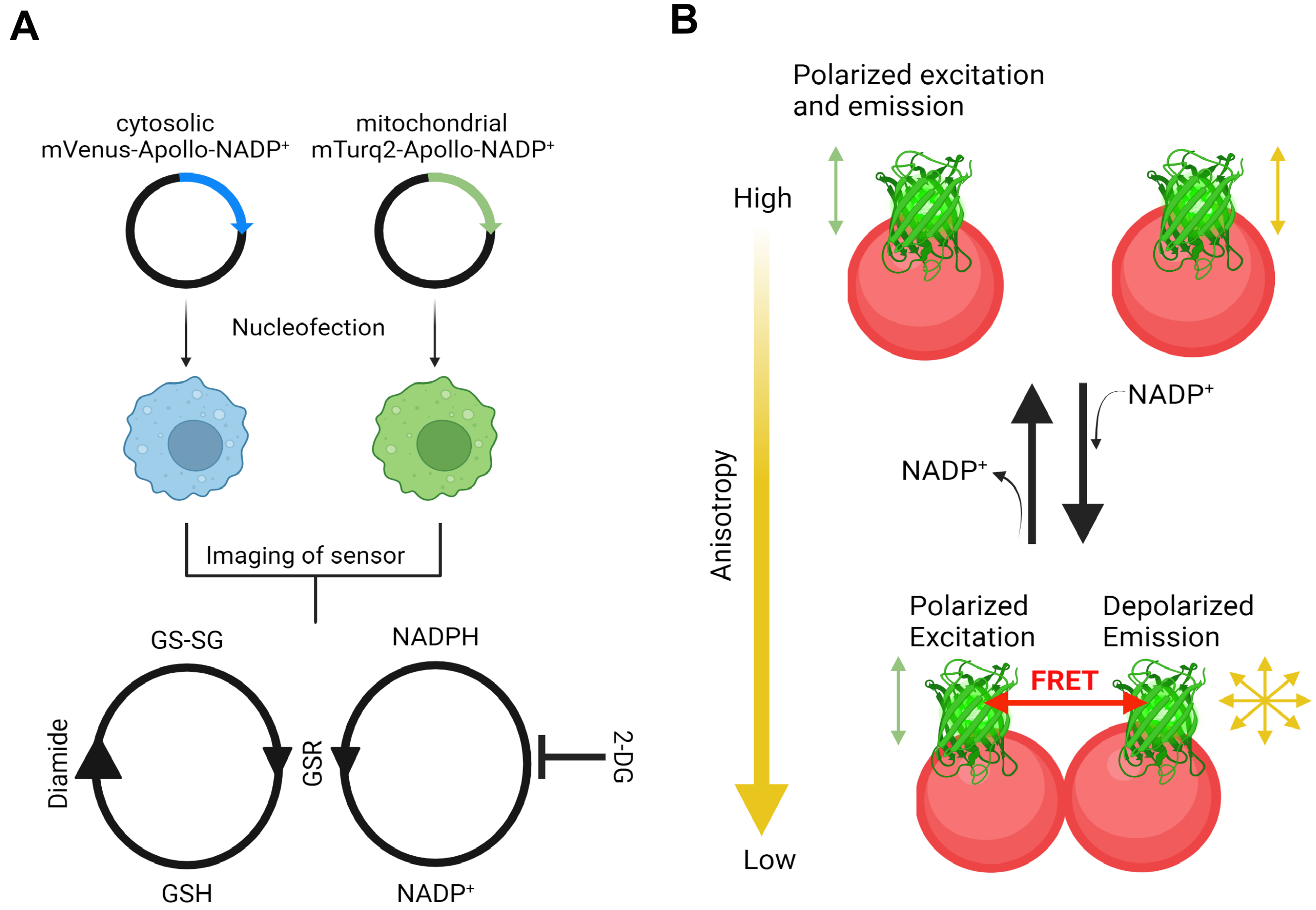

 Click here to access/download;Figure;Figure_2.pdf

Figure 2

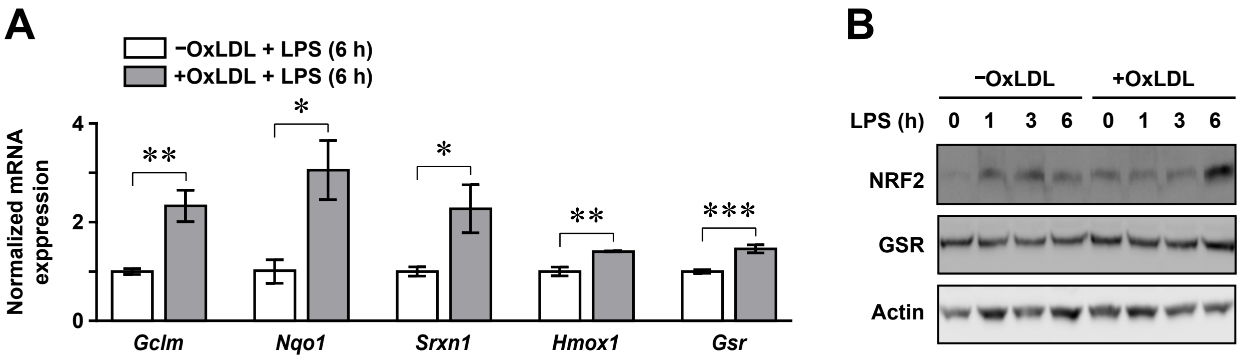

 Click here to access/download;Figure;Figure_3.pdf

Figure 3

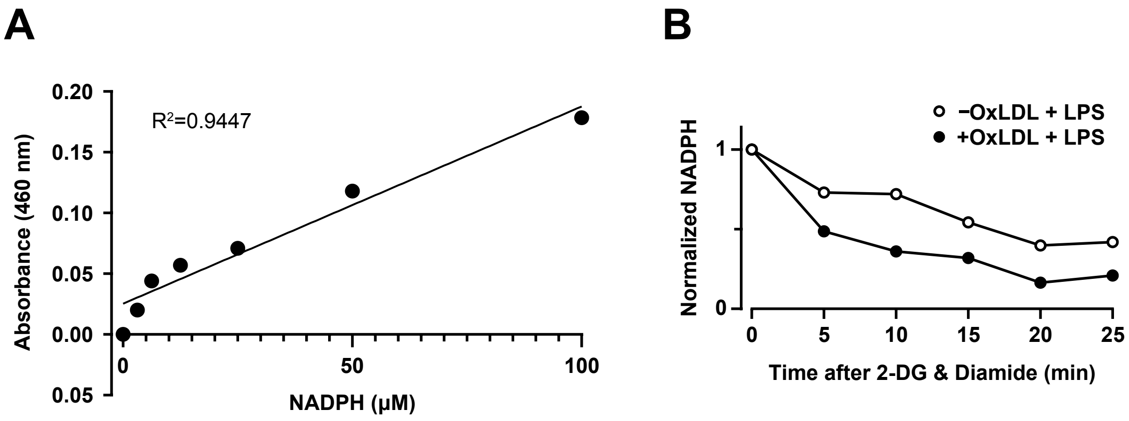

Figure 4

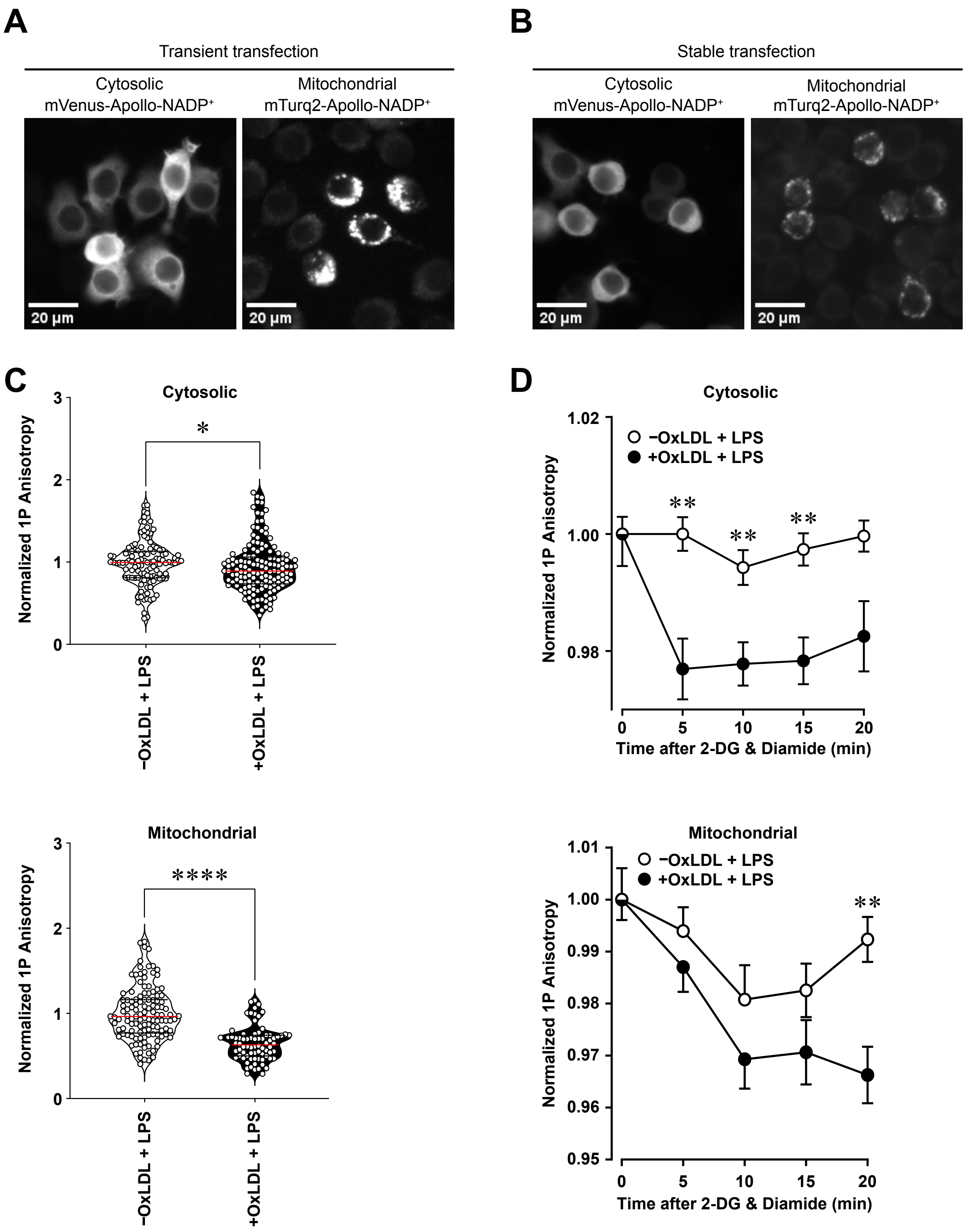
A
Transient transfection
Cytosolic
mVenus-Apollo-NADP+
Mitochondrial
mTurq2-Apollo-NADP+
20 µm
B
Stable transfection
Cytosolic
mVenus-Apollo-NADP+
Mitochondrial
mTurq2-Apollo-NADP+
20 µm
C
Cytosolic
Normalized 1P Anisotropy
*
−OxLDL + LPS
+OxLDL + LPS
Mitochondrial
****
D
Cytosolic
−OxLDL + LPS
+OxLDL + LPS
**
Time after 2-DG & Diamide (min)
Mitochondrial

Click here to access/download
Supporting Information
S1 file.docx
