# Supplementary material for: Measuring the rate of NADPH consumption by glutathione reductase in the cytosol and mitochondria
Source: PLoS One. 2024 Dec 5;19(12):e0309886. doi: 10.1371/journal.pone.0309886 (PMC11620681; doi:10.1371/journal.pone.0309886)
Supplement: Attachment — Submitted filename: Rebuttal Letter_MIC.docx [file pone.0309886.s004.docx]

**RESPONSE TO THE REVIEWERS’ COMMENTS**

I would like to thank reviewer #1 for their insightful comments (copied below in bold font). Each point that was raised is addressed below. Changes to the text in the revised manuscript are highlighted in yellow.

1. **Based on what did you use 10 ml of trypsin (usually a lot for a flask of this size)? Please describe this. What is the final concentration of trypsin? Is it necessary to cool the fugue to this cell line?**

We agree with the reviewer that 10 ml of trypsin is more than the typical recommended volume for a T75 flask. We have now adjusted the volume of both trypsin and DMEM, which deactivates the trypsin, to 3 ml (LINE 164 – 165). This volume is based on the recommendations from StemCell Technologies as we purchased the T75 flasks from this company (<https://cdn.stemcell.com/media/files/pis/29301-PIS_1_0_2.pdf>). We believe that changing the volume of trypsin will not change the results generated in our study. The final concentration of trypsin is 0.25% Trypsin/ 0.1% EDTA prior to deactivation with 10% FBS in DMEM.

We are unsure of the last question. We assume that the reviewer is asking if it is necessary to cool the centrifuge for this cell line. Our response is that centrifugation was performed at room temperature or 22 degrees C (LINE 165).

2. What conditions were used during this time?

We have now revised the manuscript and provided the recommended antibody concentration dilution in our method section, as well as the temperature of primary antibody incubation with PVDF membrane overnight (LINE 330 – 333).

3. Have you used a standard protocol for QPCR ? If not, please provide details of the final concentrations.

Yes. We have used a standard protocol for QPCR. We have now revised the manuscript to provide more details of the final concentration and the QPCR protocol (LINE 356 - 358).

4. Please clarify the item numbers of groups. Only one group has an item number description. Please specify the item numbers of the groups. What statistical software used to? Please indicate this in the main text.

We have now revised the manuscript and provided a paragraph in the method section, titled as “statistical analysis”, to clarify the definition of ‘n’ and the description of statistical software used to calculate the provided statistics (LINE 360 – 364).
